# The evolving mystery of why skeletal muscle is spared in seropositive neuromyelitis optica

**DOI:** 10.1111/jcmm.13482

**Published:** 2018-01-24

**Authors:** Alan S. Verkman, Xiaoming Yao, Alex J. Smith

**Affiliations:** ^1^ Departments of Medicine and Physiology University of California San Francisco CA USA

Dear Editor:

Autoantibodies against aquaporin‐4 (AQP4) in seropositive neuromyelitis optica spectrum disorders (called here NMO) initiate pathology in the central nervous system by binding to AQP4 on astrocytes. It has been puzzling why skeletal muscle, which also expresses AQP4, is rarely affected in NMO, despite easy access of circulating NMO autoantibody (AQP4‐IgG) to AQP4 on skeletal muscle.

Rosito *et al*. 1 report evidence that differences in the supramolecular organization of AQP4 in skeletal muscle versus brain astrocytes are responsible for the sparing of skeletal muscle in NMO, arguing that the size of AQP4 supramolecular clusters (called orthogonal arrays of particles, OAPs) is smaller in skeletal muscle than in astrocytes resulting in reduced AQP4‐IgG binding. This mechanism is motivated by the observation that some AQP4‐IgG autoantibodies bind better to AQP4 OAPs than to separated AQP4 tetramers 2 and that AQP4 OAPs are required for C1q binding and complement activation 3. Although the study of Rosito *et al*. 1 is a novel and earnest attempt to solve the puzzle of skeletal muscle sparing in NMO, in our opinion their explanation lacks theoretical plausibility and contradicts available data.

The authors’ assertion that arrays are smaller in muscle than in brain is based on biochemical analysis and super‐resolution (STED) microscopy. Non‐denaturating gels showed minor differences in the size of AQP4 aggregates between muscle and brain; however, the ratio of M1‐AQP4 to M23‐AQP4 was 1:3 or less in both tissues and in the range where large OAPs form, which can bind all AQP4‐IgG autoantibodies in heterologous systems 2. As OAPs in native tissues are small (20‐50 nm diameter) and tightly packed, detecting individual arrays is a challenge even for super‐resolution microscopy and requires careful consideration of microscope sensitivity to cluster size and density 4. Freeze‐fracture electron microscopy, the gold standard in studying AQP4 OAP size, has shown that OAPs in healthy human skeletal muscle consist of 10‐35 AQP4 tetramers spaced 6 nm apart 5, similar to the size of OAPs in brain 6; Rosito *et al*. 1, however, report cluster size an order of magnitude greater.

Even if OAPs were smaller in skeletal muscle than in astrocytes, little difference in AQP4‐IgG binding is expected for a ratio of M23‐AQP4 to M1‐AQP4 more than 1, as has been shown experimentally and by mathematical modelling 7. Conceptually, an AQP4‐IgG molecule bound anywhere within an AQP4 cluster away from the cluster edge cannot sense cluster size. The same consideration holds for complement activation. In the light of this, the data in Figure 1 of Rosito *et al*. 1 showing minimal or non‐specific AQP4‐IgG binding to skeletal muscle in seropositive NMO are perplexing, as other studies have shown that systemically administered AQP4‐IgG rapidly binds to rodent skeletal muscle 8 and that IgG binding is found on skeletal muscle in humans with NMO myositis 9. One potential explanation for this discrepancy might be loss of AQP4 antigenicity for autoantibody binding in the non‐fixed frozen muscle used by Rosito *et al*. 1.

If AQP4 cluster size does not account for sparing of skeletal muscle in NMO, then what does? One possibility is complement inhibitor protein CD59, which is strongly expressed in skeletal muscle and other AQP4‐expressing tissues in the periphery such as kidney and stomach. We found marked skeletal muscle injury in AQP4‐IgG seropositive rats deficient in CD59, with creatine phosphokinase levels nearly 1000‐fold over normal 10. Perhaps the very rare patients with NMO myositis have CD59 polymorphisms. Another possibility, though speculative, is that unique environmental factors in the central nervous system, such as the presence of microglia, a narrow extracellular space and the close proximity of AQP4 clusters to blood vessels, may amplify subthreshold injury following AQP4‐IgG binding to astrocyte AQP4. Differences in astrocyte versus skeletal muscle biology may also be involved.

The mystery continues!

## Conflict of interest

The authors declare that they have no conflict of interest to disclose.
